# Seed extract of Thai *Mucuna pruriens* reduced male reproductive damage in rats induced by chronic stress

**DOI:** 10.1080/13880209.2022.2034896

**Published:** 2022-02-18

**Authors:** Natthapol Lapyuneyong, Nareelak Tangsrisakda, Pannawat Choowong-In, Kowit Chaisiwamongkol, Nongnut Uabundit, Tarinee Sawatpanich, Supatcharee Arun, Alexander Tsang-Hsien Wu, Sitthichai Iamsaard

**Affiliations:** a Department of Anatomy, Faculty of Medicine, Khon Kaen University, Khon Kaen, Thailand; b Research Institute for Human High Performance and Health Promotion (HHP & HP), Khon Kaen University, Khon Kaen, Thailand; c College of Medical Science and Technology, The PhD Program for Translational Medicine, Taipei Medical University and Academia Sinica, Taipei, Taiwan; d Graduate Institute of Medical Sciences, National Defense Medical Center, Taipei, Taiwan

**Keywords:** Tyrosine phosphorylated protein, caspase, androgen receptor, testis, sperm

## Abstract

**Context:**

Thai *Mucuna pruriens* (L.) DC. var. *pruriens* (Fabaceae) (TMP) is known to enrich reproduction but preventive effects on stress related adverse reproductive parameters are not documented.

**Objective:**

This study investigates the protective property of TMP seed extract on reproductive damage under chronic stress (CS).

**Materials and methods:**

Male Sprague-Dawley rats were divided into four groups. The control and CS groups received distilled water, whereas the pre-treated rats received the aqueous TMP seed extract at doses of 150 and 300 mg/kg BW for 20 days before co-treatments with CS induction (immobilization and forced swimming) for 81 days. Serum was used to determine the cortisol and testosterone levels. Histology of testis and epididymis was observed with localization of androgen receptor (AR). Sperm parameters and the expression of steroidogenic acute regulatory (StAR), cytochrome P450 family 11 subfamily a member 1 (CYP11A1), AR, HSP70, caspases (3 and 9) and tyrosine phosphorylation (TyrPho) proteins were investigated.

**Results:**

TMP extract improved cortisol level (0.84 ± 0.02 µg/dL) and protected against the damage of reproductive tissues and sperm parameters (count [49.78 ± 3.74 million sperm/mL], viability [90.01 ± 1.17%] and precocious acrosome reaction [1.38 ± 0.48%]). Expression of testicular StAR, CYP11A1, AR and HSP70 proteins was improved. Caspase expression was decreased in treated rats. TMP increased AR expression in CS sperm. Moreover, TyrPho protein expression was corrected after TMP administration.

**Conclusions:**

TMP seed protected against adverse reproductive parameters in CS via improvements of functionally testicular markers and reductions of apoptotic proteins. It is possible to develop the TMP beans as an alternative medicine in treating of male subfertility caused by CS.

## Introduction

Chronic stress (CS) is known to not only cause neurological and vital organ function disorders but also leads to infertility in both animals and humans. Basically, stress can interrupt the hypothalamus pituitary adrenal axis, resulting in significant increase of serum cortisol levels (Koolhaas et al. 1997; Hannibal and Bishop 2014; Fogelman and Canli 2018). Consequently, the excess cortisol hormone caused by CS has been shown to suppress male sex hormones such as gonadotropin-releasing hormone (GnRH), luteinizing hormone (LH), follicular-stimulating hormone (FSH) and testosterone as previously described (Herman 2013; Lin et al. 2014; Mohamadpour et al. 2017). Indeed, CS could affect male sexual behaviours (Grønli et al. 2005; Quintana et al. 2018; Choowong-In et al. 2021). In addition, it has been reported that CS causes male infertility by damaging of reproductive parameters including testicular functions and structures, epididymis histology and sperm qualities (Nirupama et al. 2013; Prabsattroo et al. 2015; Qiu et al. 2019).

Functionally, the CS rats have been shown to have lower expression of steroidogenic acute regulatory (StAR), cytochrome P450 family 11 subfamily a member 1 (CYP11A1) and tyrosine phosphorylation (TyrPho) proteins in testicular lysate (Arun et al. 2016). Such proteins are involved in testosterone biosynthesis. In addition, it was demonstrated that the androgen receptor (AR) expression in the germinal epithelial cells including Sertoli cells was significantly decreased in stress animals (Türk et al. 2015; Roboon et al. 2016; Thanoi et al. 2018). Moreover, stress caused from heat induction could induce apoptosis and decrease of AR expression in animal testis (Türk et al. 2015). Although AR was lower expressed in acute stress testis (Roboon et al. 2016), both AR and Hsp70 (heat shock protein 70) expressions were increased in testis induced chronic heat stress (Shen et al. 2019). Notably, the features of CS that differentiate it from acute stress are more depression, increased cortisol level, decreased testosterone level and changes of functional proteins of testis. Currently, many side effects of drugs used for treating neurological diseases, including stress, have been reported (Schiavi et al. 1997; Moskovic et al. 2011). Therefore, alternative medicines such as plant extracts that have fewer side effects are being searched.

As documented, many potential plant extracts such as *Phyllanthus emblica* L. (Phyllanthaceae) leaf, *Moringa oleifera* Lam. (Moringaceae) leaf and *Mucuna pruriens* (L.) DC. var. *pruriens* (Fabaceae) seed were shown to improve the male reproductive damages caused by stress (Prabsattroo et al. 2015; Arun et al. 2018; Ashidi et al. 2019). The seed of *Mucuna pruriens* (MP), a miracle bean commonly used in Indian traditional medicine, has been reported to have various phytochemical substances (Agbafor and Nwachukwu 2011; Nwaoguikpe et al. 2011; Anosike et al. 2019). In addition, the major substance reported in MP seeds is l-DOPA stimulating GnRH secretion, resulted in increased sex hormones (Vermes et al. 1979; Misra and Wagner 2004; Shukla et al. 2009; Singh et al. 2013; Mutwedu et al. 2019). Moreover, such seeds have been demonstrated to improve the male reproductive dysfunction caused by stress (Ashidi et al. 2019).

Thai *Mucuna pruriens* (TMP) is botanically different from Indian species in possessing histamine activity in spines of the seed pods. Traditionally, TMP seed has been used to enhance aphrodisiac activity for older men with low libido. However, the scientific support for such property is still limited. Recently, TMP seed has been shown to possess high antioxidant capacity and to increase the expressions of testicular proteins (AR, AKAP4 [A-kinase anchor protein 4] and TyrPho proteins), testosterone level and sperm concentration in healthy rats (Iamsaard et al. 2020). Since TMP seeds seemed to enrich male reproductive system and its protective effect in stress animal models has never been reported, this study investigates improvement of the seed extract on reproductive damage in male rats induced by CS.

## Materials and methods

### Preparation of TMP seed extract

The powder of TMP aqueous seed extract used in this study was kindly obtained from Dr. Sitthichai Iamsaard’s Laboratory. The voucher specimen (no. S. Iamsaard 01) was collected in the herbarium database, Department of Biology, Khon Kaen University. The seed extract had been previously demonstrated to have antioxidant capacity and total phenolic compounds with no toxicity on male reproductive system (Iamsaard et al. 2020). Recently, this TMP extract has been already proven by our research group to contain l-DOPA determined by NMR and HPLC analyses (Choowong-In et al. 2021). The TMP seed powder was prepared in the concentrated stock with DW before diluting to be 150 and 300 mg/BW of each animal in treated groups. The selected doses used in this study were based on its property to increase the testosterone level, sperm quality and essential testicular proteins as previously demonstrated (Iamsaard et al. 2020).

### Animals, experimental design and stress induction

The 32 adult male Sprague-Dawley rats (270–300 g) were purchased from the Siam Nobura Corporation, Pathumwan, Bangkok, Thailand. All rats were kept in plastic cages under the experiment room condition (12 h light/dark cycle, temperature 23 ± 2 °C and humidify 30–60% RH, relative humidity) at Laboratory Animal Unit, Faculty of Medicine, Khon Kaen University, Thailand. Animals received pellet food and water *ad libitum* during acclimatization for seven days. Then, rats were divided into control, CS, CS + T-MP150 and CS + T-MP300 groups. In pre-treatment period (days 1–20), the control and CS animals received distilled water (DW) while TMP treated rats received TMP extract at doses of 150 and 300 mg/kg/day solved in DW via oral gavage. For co-treatment period (60 days), the control and CS groups were administered DW whereas TMP treated animals received the extract before immobilization within plastic cylinder tube (4 h) and followed by forced swimming at 15 °C (15 min) as previously described by Arun et al. (2018).

### Animal ethics

This study has been approved by the Animal Ethics Committee of Faculty of Medicine, Khon Kaen University, based on the Ethic of Animal Experimentation of National Research Council of Thailand (code: AEMDKKU 012/2019).

### Hormone analyses and sample collection

At the end of experiment, animals were anaesthetized by thiobarbital sodium (60 mg/kg BW via intraperitoneal injection) and euthanized by cervical dislocation (Iamsaard et al. 2020). Then, the blood was collected from the left ventricle by cardiac puncture. The drawn blood was centrifuged at 4 °C, 13,000 rpm for 15 min to separate the serum from the blood cells (Iamsaard et al. 2020; Chaimontri et al. 2021). After that, the testosterone and cortisol hormone levels were analysed from the blood serum at the Radiology Unit, Srinagarind Hospital, Faculty of Medicine, Khon Kaen University, Thailand. After the blood collection, the testes, epididymis plus vas deferens, and penis were rapidly collected and their fat pads surrounding tissues were removed before weighed and recorded. Then, the penis, right testis and right epididymis plus vas deferens were fixed in 10% formalin for histological examinations. To investigate essential protein expression, the left testis and epididymal sperm pellet were snap-frozen in liquid nitrogen and kept at −20 °C before extraction.

### Morphological study

The fixed testis and caudal epididymis were further routinely processed for making of paraffin tissue blocks before sectioning by semi-autonomic rotary microtome (ERM3100 Hestion Histology Equipment) at 5–7 µm thickness. After deparaffinization, all tissue sections were stained with haematoxylin (Sigma-Aldrich, St. Louis, MO) and eosin (Sigma-Aldrich, St. Louis, MO). The histopathology of tissues was observed and photographed under light microscope (Nikon light ECLIPSE E200 microscope equipped with a DXM1200 digital camera, Tokyo, Japan). The sperm mass within epididymal lumen was investigated to compare with sperm concentration. For morphometric analysis, the seminiferous tubules (50 tubules per animals) were measured for diameter (four axes/tubule, between opposite side of myoid cells) and germinal epithelial height (eight sides/tubule, from basement membrane to adluminal surface). All parameter lengths were analysed by using the ImageJ program [ver. 1.50i; National Institutes of Health (NIH), Bethesda, MD].

### Evaluation of sperm quality analyses

Epididymal sperm mass and fluid were retrieved from vas deferens and caudal epididymis by squeezing into 1 mL of fresh PBS (30 °C and pH, 7.4). After resuspension, the stocked epididymal sperm solution was aliquoted (50 µL) to further examine the sperm viability. Then, the epididymal sperm suspension (150 µL) was aliquoted as a stocked solution to be fixed with 4% paraformaldehyde, pH 7.4, at 4 °C for further examination of the sperm concentration, morphology and precocious acrosome reaction.

### Sperm viability

Sperm suspension (50 µL) was mixed and incubated with 50 µL of eosin-nigrosin solution (BASO Diagnostics, Inc., Zhuhai, China) under 30 °C for 40 s (Chaimontri et al. 2021). Then, 10 µL stained sperm solution was dropped and smeared onto glass slide in triplicates. Total viable and dead sperm (400 cells) were evaluated under light microscope. As observed, the dead sperm shows pink colour of the eosin while the live sperm shows no staining. The sperm viability was calculated as percentage of total sperm.

### Sperm concentration

Sperm suspension (20 µL) prepared from stock above was loaded onto haemocytometer chamber. Under light microscope, the sperm were counted within two upper and lower counting chambers in triplicate examinations and calculated to be sperm concentration (million cells/mL) as previously described (Iamsaard et al. 2020; Tangsrisakda and Iamsaard 2020).

#### Sperm morphology

Sperm suspension (20 µL) was smeared on glass slide and then fixed with methyl alcohol for 20 min. Subsequently, fixed sperm was stained with 1% eosin (Sigma-Aldrich, St. Louis, MO) for 10 min to observe the normal and abnormal sperm cells (Iamsaard et al. 2020; Chaimontri et al. 2021). A total of 400 sperm cells of each rat were observed under light microscope and classified as normal and abnormal sperms. The abnormal sperm morphologies include head, tail and multiple abnormal sperms. All abnormal sperms were calculated as percentages of sperm abnormality.

### Acrosome reaction status assay

The fixed sperm suspension (10 µL) was diluted with PBS (1:10). Then, the diluted sperm (10 µL) was dropped and smeared on coated glass slides in duplicates. The dried sperm on slides were re-fixed with 4% paraformaldehyde for 15 min and further stained with Coomassie Brilliant Blue G 250 (0.22%, Bio-Rad Laboratories, Inc., Hercules, CA) for 5 min (Iamsaard et al. 2020; Yannasithinon et al. 2021). Then, the acrosome statuses (acrosome intact (AI) sperm and acrosome reacted (AR) sperm) were observed at head of total 400 sperms under light microscope. The AI sperm is obviously stained with Coomassie blue at acrosomal cap while the AR sperm has no staining.

### Immunofluorescence analysis

Testicular sections were deparaffinized with xylene and rehydrated with descending serial alcohols and DW. After that, deparaffinized sections were soaked in 1× citrate buffer (three times) under microwave at 300 W. Then, they were permeabilized by incubating with 0.2% Triton X-100 for 10 min in a moist chamber. To prevent the collapsing of antibody solution, the boundary of tissues on glass slide was circled by PAP pen (5 mm; Millipore Co., Billerica, MA) for immunostaining. After that, the non-specific proteins were blocked with 3% bovine serum albumin (BSA) in PBS. Then, the sections were incubated with primary AR antibody (1:100 dilution; Santa Cruz Biotechnology, Inc., Dallas, TX) in a moist chamber for overnight while the g for the primary antibody. After washing unbound primary antibody, the antigen-primary antibody complexes and negative control slides were probed with secondary anti-mouse antibody (1:300 dilution; Alexa Flour^®^ 488) for 90 min. Then, all slides were nuclear counterstained with Hoechst dye (1:10,000 dilution; ab228551, Abcam, Cambridge, UK) to allow DNA binding for 5 min before being mounted with glycerol. Under fluorescence microscope (Nikon ECLIPSE 80i, Tokyo, Japan), the positive AR expression was emitted as green fluorescence by using fluorescein 5-isothiocyanate (FITC) filter while the 4′,6-diamidino-2-phenylindole (DAPI) emission filter was used to locate nuclei emitting as blue fluorescence.

### Immuno-Western blotting analysis

To prepare the total protein lysate, testis or sperm pellet was extracted with 1× RIPA (radioimmune precipitation assay) buffer (Cell Signaling Technology Inc., Boston, MA) containing protease inhibitor cocktails (Sigma-Aldrich, Inc., St. Louis, MO). After that, the sample lysates were homogenated on ice and further sonicated with the ultrasonic processor (20 Hz, 60 pulses; Cole, Parmer, Vernon Hills, IL). The homogenated samples were centrifuged at 4 °C, 14,000 rpm for 10 min to separate a pellet from the supernatant containing total protein lysate. Then, the supernatant was measured for a total protein concentration (µg/µL) using a spectrophotometer (ND-1000; Manual Nano Drop V3.5 User’s Technology Inc., Wilmington, DE).

### Detections of testicular and sperm protein expressions

To investigate changed expression of AR, caspase 3, caspase 9, CYP11A1, HSP70, StAR and TyrPho proteins in testicular and sperm lysates, total protein separations and western blotting were performed. Total loading samples (80 µg/lane) of each group was loaded and separated on 10% sodium dodecyl sulphate (SDS) polyacrylamide gel by electrophoresis (PAGE). Then, separated proteins were transferred onto nitrocellulose membranes at 110 V, 3 h. In sequence, the transferred proteins were blocked for non-specific bindings with 3% skim milk (3 g of skim milk in 10 mL of 0.1% PBS, pH = 7.4) for 45 min. The protein membranes were further incubated with specific primary antibodies including anti-AR, StAR, CYP11A1, TyrPho, caspase 3, caspase 9 or Hsp 70 (1:2000 dilution; Santa Cruz Biotechnology, Inc., Dallas, TX) at 4 °C, overnight while anti GAPDH was used as internal control. After that, the non-binding antibodies were washed with 0.05% PBST three times (5 min for each time). Then, the membrane for StAR detection was incubated with secondary antibody (goat anti-rabbit, 1:1,000 dilution; Santa Cruz Biotechnology, Inc., Dallas, TX) while those membranes for AR, CYP11A1, TyrPho, caspase 3, caspase 9, Hsp 70 and GAPDH were incubated with secondary antibody (goat anti-mouse, 1:2000 dilution; Santa Cruz Biotechnology, Inc., Dallas, TX) at room temperature for 2 h. All membranes were washed for unbound secondary antibodies with 0.05% PBST three times (5 min for each time). To detect positive protein band, each membrane was incubated with ECL (enhanced chemiluminescence) substrate reagent kit (GE Healthcare Life Sciences, Piscataway, NJ) and visualized under the gel doct apparatus (ImageQuant 400, GE Healthcare, Pittsburgh, PA).

### Statistical analysis

All data were expressed as mean ± standard deviation (SD). To compare the difference between four groups, all parameters were analysed by using one-way ANOVA (post hoc, LSD and descriptive) test from the IBM SPSS statistic program (version 22, Armonk, NY), downloaded from KKU software center, Khon Kaen University, Thailand (November 2019). If the *p* value was less than 0.05, the difference was considered as statistical significance.

## Results

### Effects of TMP extract on gross morphology of CS reproductive organs

**Figure 1.**
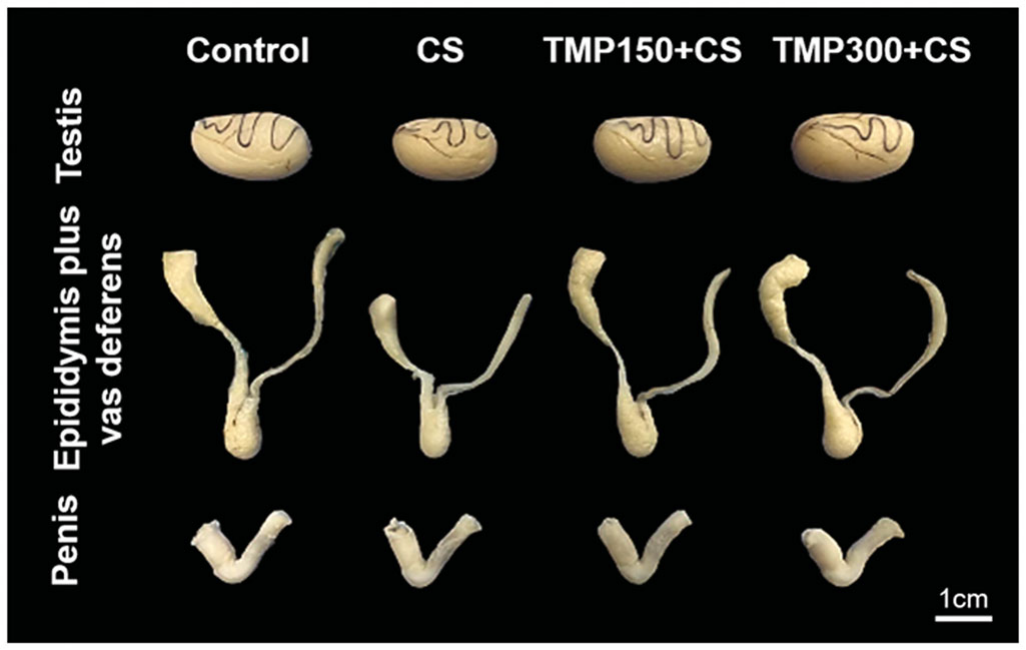
Representative photographs comparing gross morphology of testes, epididymis plus vas deferens, and penis of control, CS, TMP150 + CS and TMP300 + CS groups in administration for consecutive 81 days. CS: chronic stress; TMP: Thai *Mucuna pruriens*.

As shown in Figure 1, the testis and epididymis plus vas deferens in CS group were obviously smaller than those of control group. It was found that the sizes of such organs were improved in TMP treated groups. However, no difference in penis morphology was observed as compared among control and TMP treated groups (Figure 1).

### Effects of TMP extract on the weights and reproductive parameters in CS rats

This study found that the percent of changed body weights of TMP + CS groups were significantly improved as compared to CS group (Table 1). In addition, the relative weights of testis and epididymis plus vas deferens in TMP treated groups were significantly increased as compared to CS group. In contrast, both absolute and relative weights of penis were not different among groups. Significantly, it was revealed that the seminiferous tubule diameter and epithelial height of TMP treated groups were increased in dose dependence compared to those of CS rats (Table 1).

**Table 1.** The body weight and reproductive parameters compared among control, CS, TMP150 + CS and TMP300 + CS treated groups.

Parameters	Groups			
	Control	CS	TMP150 + CS	TMP300 + CS
Percent changed body weight (%)	115.01 ± 4.18	47.62 ± 8.08*	72.54 ± 9.32^#^	65.47 ± 9.91^#^
Final body weight (g)	603.20 ± 67.05	470.79 ± 40.51*	532.41 ± 41.61^#^	497.29 ± 31.08
Testis				
Absolute weight (g)	1.909 ± 0.116	1.715 ± 0.179*	1.741 ± 0.051	1.811 ± 0.116
Relative weight (g/100 g BW)	0.305 ± 0.013	0.291 ± 0.106	0.335 ± 0.015	0.377 ± 0.049^#^
Epididymis plus Vas deferens				
Absolute weight (g)	0.916 ± 0.076	0.666 ± 0.079*	0.810 ± 0.065^#^	0.807 ± 0.041^#^
Relative weight (g/100gBW)	0.144 ± 0.010	0.149 ± 0.024	0.147 ± 0.024	0.166 ± 0.020^#^
Penis				
Absolute weight (g)	0.371 ± 0.025	0.350 ± 0.029	0.363 ± 0.018	0.379 ± 0.041
Relative weight (g/100 g BW)	0.062 ± 0.007	0.074 ± 0.003	0.069 ± 0.008	0.081 ± 0.015
Seminiferous tubules				
Diameter (µm)	307.10 ± 12.28	243.70 ± 34.96*	312.38 ± 13.81^#^	346.23 ± 17.71^#^
Epithelial height (µm)	81.55 ± 6.82	62.79 ± 6.87*	85.57 ± 6.86^#^	90.61 ± 6.85^#^

CS: chronic stress; TMP: Thai *Mucuna pruriens L. DC var. pruriens.* Seeds extract.

Data were represented as mean ± standard deviation (SD).

* Significant difference (*p*< 0.05), compared to the control group.

# Significant difference (*p*< 0.05), compared to the CS group.

### TMP seed extract reduced the damage of testis and epididymis of CS rats

**Figure 2.**
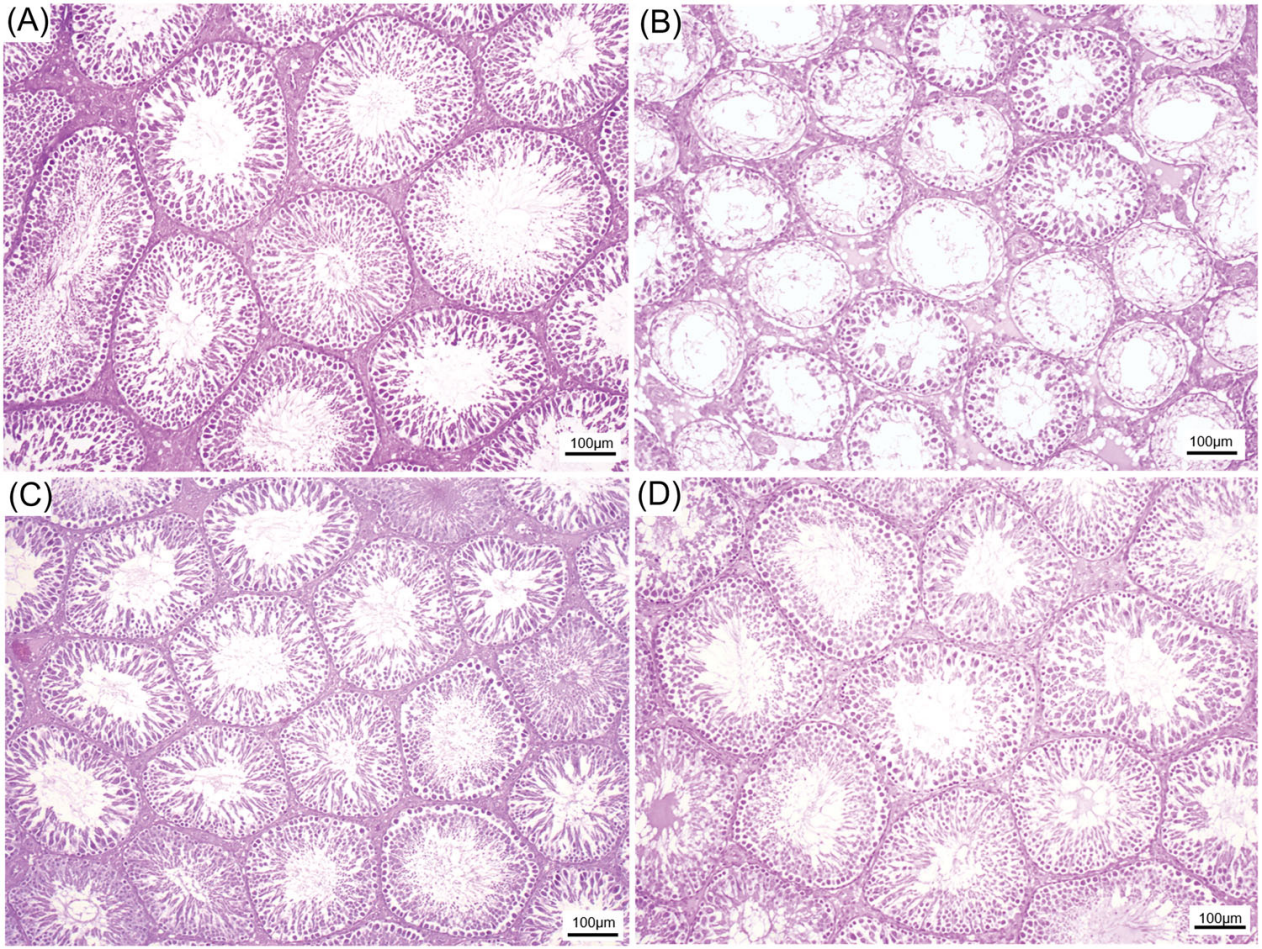
Representative histological photographs of the seminiferous tubules compared among control (A), CS (B), TMP150 + CS (C) and TMP300 + CS (D), respectively.

**Figure 3.**
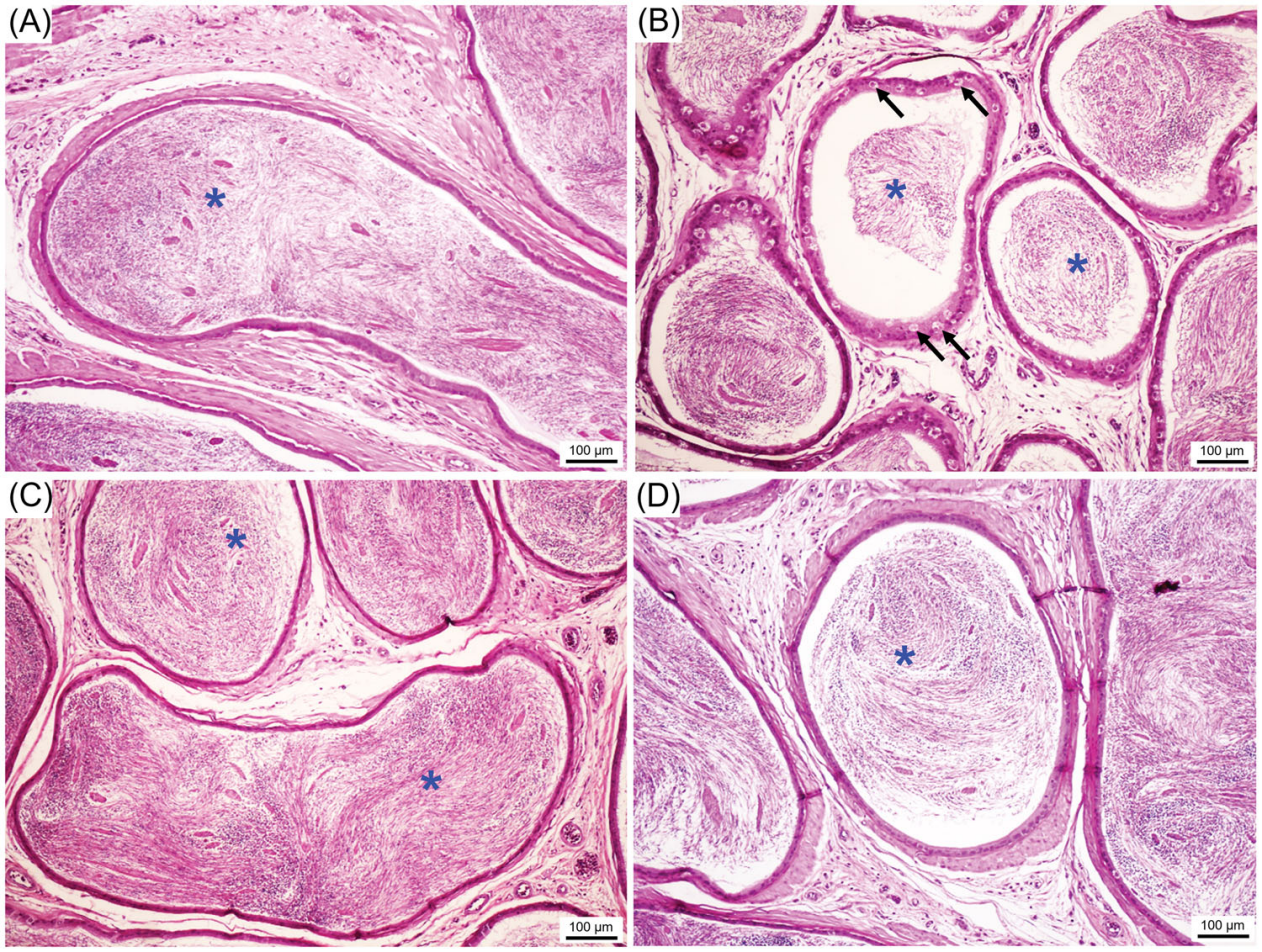
Representative histological photographs of the caudal epididymis compared among control (A), CS (B), TMP150 + CS (C) and TMP300 + CS (D). Blue asterisks: sperm mass, black arrows: hyperplasia of clear cells.

As shown in Figure 2(A,B), the CS could damage the germinal epithelium of seminiferous tubules by reductions of its diameter and germinal epithelial height as compared to that of control. Obviously, two doses of TMP extracts can reduce the testicular damage induced by CS (Figure 2). The histological features of caudal epididymis from control and treated groups are shown in Figure 3. It was observed that the sperm mass of CS group (Figure 3(B)) was decreased when compared with control group (Figure 3(A)). In addition, the hyperplasia of clear cells was found in CS group. Interestingly, the sperm mass was markedly increased in TMP + CS groups when compared with CS group (Figure 3(B–D)).

### Effects of TMP extract on corticosterone and testosterone levels

**Figure 4.**
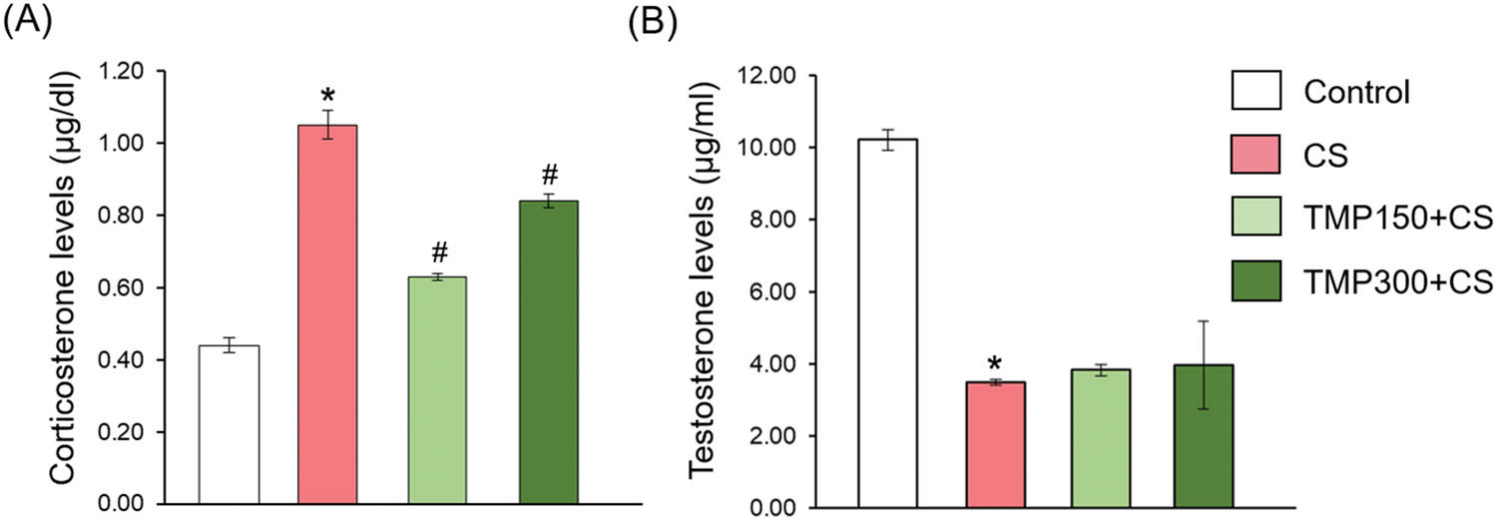
Showing the corticosterone (A) and testosterone levels (B) in the blood serum of control, CS, TMP150 + CS and TMP300 + CS in treating for consecutive 81 days. *Significant difference (*p*< 0.05), compared to control group, ^#^significant difference (*p* < 0.05), compared to CS group.

Figure 4 shows the levels of serum corticosterone and testosterone compared between control, CS and TMP treated groups. In CS group, the corticosterone levels were significantly increased when compared with control group. Both doses of TMP extracts could improve such levels in CS group (Figure 4(A)). In contrast, although CS significantly decreased the testosterone levels, TMP extracts did not improve such hormone in CS animals (Figure 4(B)).

### Effect of TMP seed extract on improvement of sperm qualities

**Figure 5.**
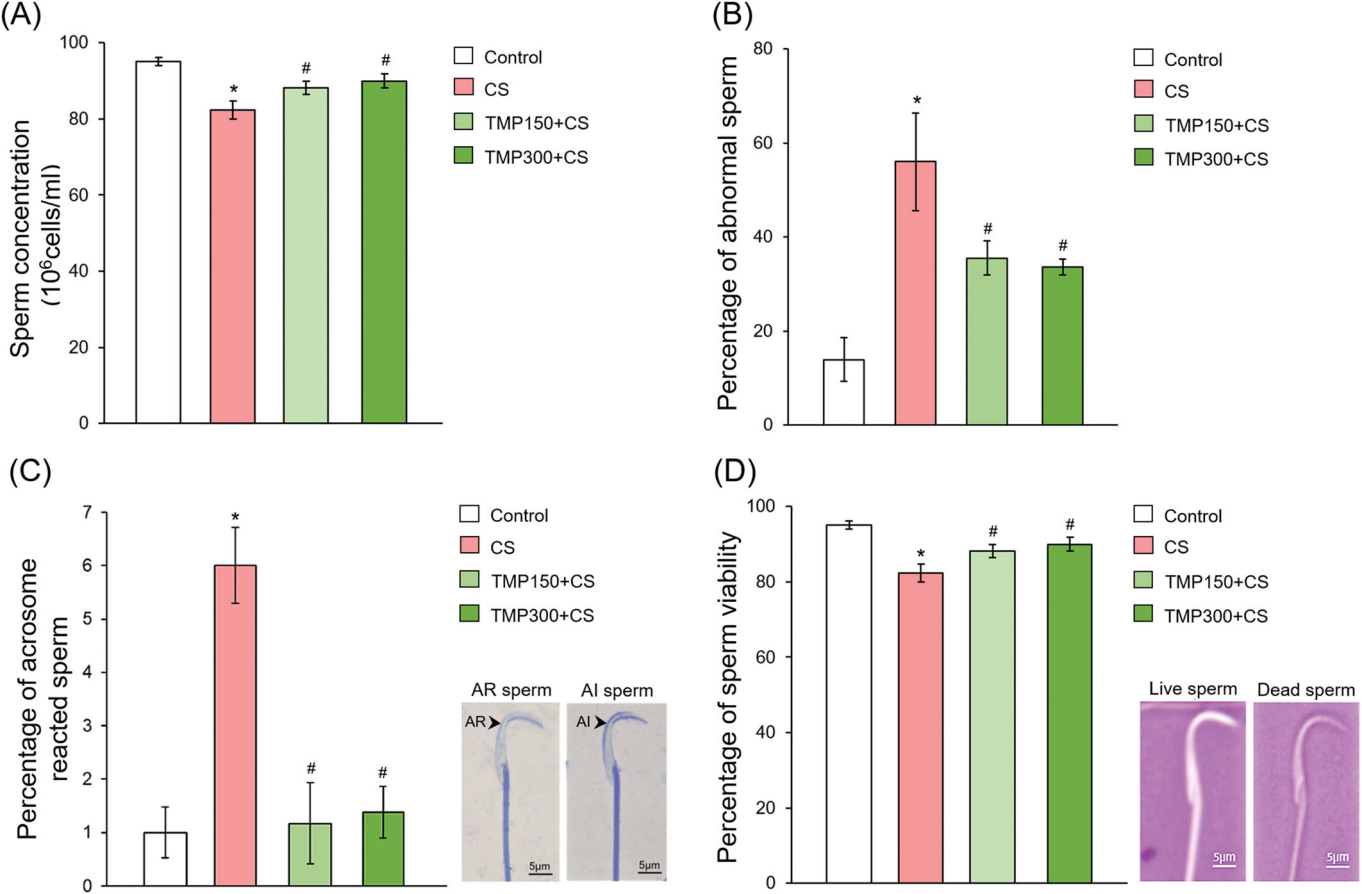
Percentage of sperm concentration (A), abnormal sperm (B), acrosome reacted (AR) sperm (C) and sperm viability (D) compared among control, CS, TMP150 + CS and TMP300 + CS groups. AR: acrosome reacted; AI: acrosome intact stained by 0.22% Coomassie blue; dead sperm stained by eosin-nigrosin dyes. *Significant difference (*p*< 0.05), compared to control group, ^#^significant difference (*p* < 0.05), compared to CS group.

The sperm quality retrieved from the epididymis of all groups is shown in Figure 5. It was found that sperm concentration in CS group was significantly decreased when compared with control group (Figure 5(A)). Interestingly, TMP extracts could significantly increase the sperm concentration as compared to CS group (Figure 5(A)). In addition, CS increased the percentage of sperm morphological abnormality which was improved when treated with TMP seed extracts (Figure 5(B)). The right panel of Figure 5(C) shows the sperm acrosome statuses evaluated in this study consisting of the AR and AI sperms. The percentage of AR sperm of CS group was significantly increased when compared with control group and it was improved after treating with TMP administration (Figure 5(C)). The sperm viabilities are shown as unstained live sperm (right panel of Figure 5(D)). It was found that the percentage of sperm viability in TMP groups was significantly increased as compared with CS group (Figure 5(D)).

### Androgen receptor expression in control and CS testes

**Figure 6.**
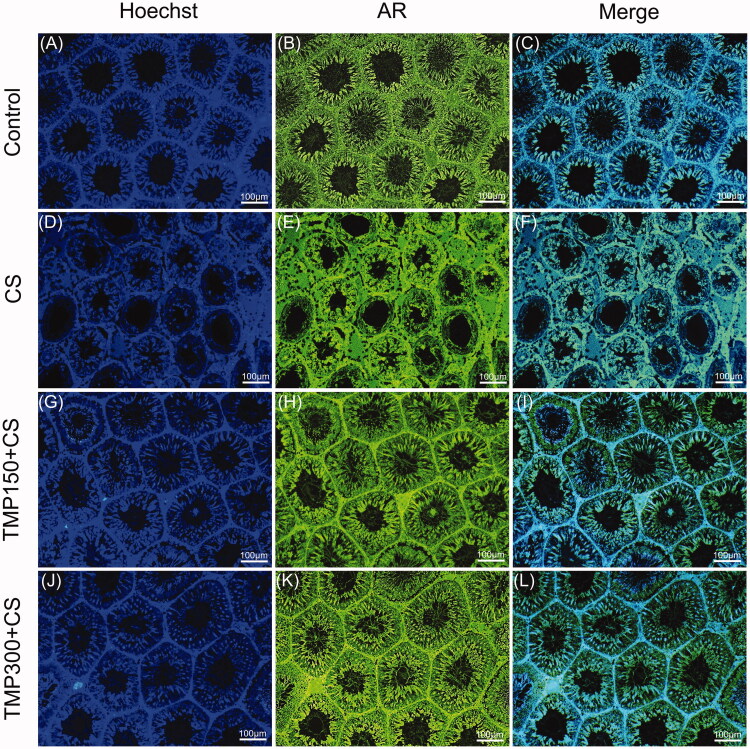
The immunofluorescence micrographs showing localization of androgen receptor (AR, green) merged with Hoechst in rat testicular tissue compared among control (A–C), CS (D–F), TMP150 + CS (G–I) and TMP300 + CS (J–K) groups. Hoechst 33342 was used as a nuclear counter stain (blue).

As shown in Figure 6, the AR was generally localized in interstitial tissues and germinal epithelium of seminiferous tubules in the control, CS and TMP treated groups. The different intensity of testicular AR expression was not obviously observed. It seemed that AR intensity was increased in the intertubular areas of CS groups as compared to other groups (Figure 6).

### Effects of TMP extract on changes of functionally testicular and sperm proteins

**Figure 7.**
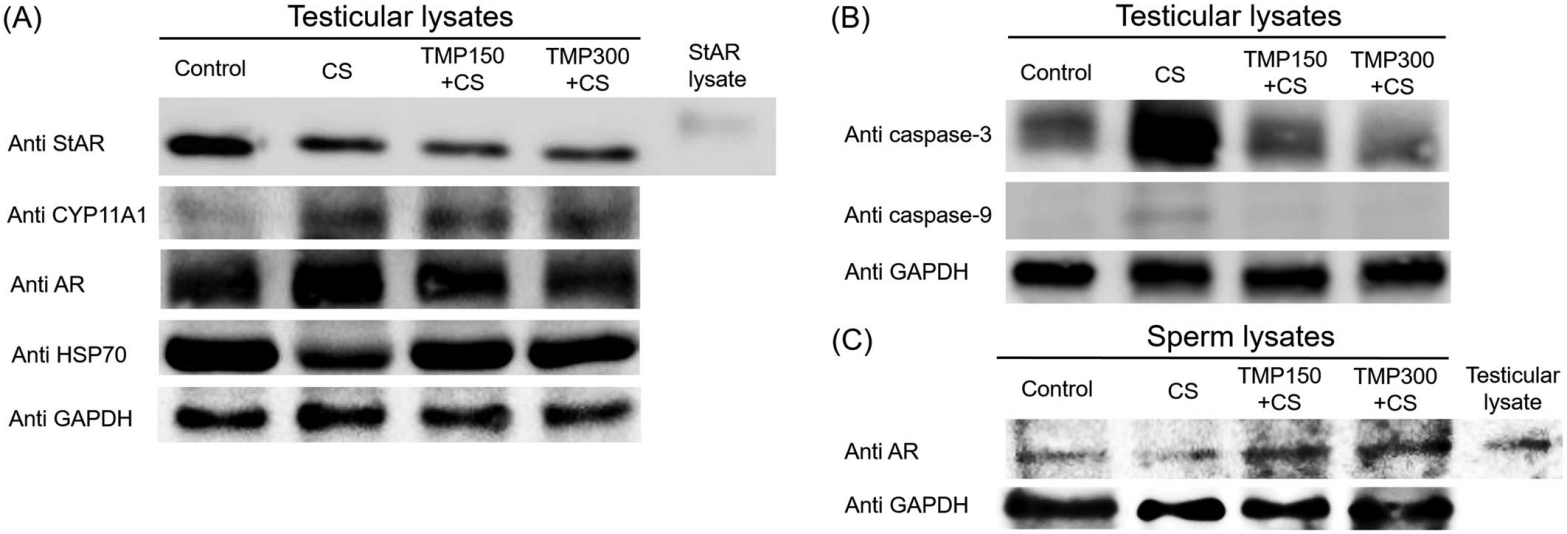
Expression of StAR (steroidogenic acute regulatory protein), CYP11 (cytochrome P450 family 11 subfamily a member 1), AR (androgen receptor protein), HSP70 (heat shock protein 70), cleaved caspase-3 and -9 compared among control, CS, TMP + CS groups, respectively. StAR lysate was used as a positive control for testicular StAR lysate. Testicular lysate was used as a positive control for sperm AR detection. GAPDH was used as an internal control.

The expression of testicular StAR protein was markedly decreased in CS group when compared with control group and it was not improved in TMP treated groups (Figure 7(A)). It was revealed that the CYP11A1 protein expression in TMP treated testes was obviously increased as compared to that of CS group as shown in Figure 7(A). Additionally, the expression of AR in CS group was increased while that of HSP70 was obviously decreased when compared to other groups (Figure 7(A)). For apoptotic markers, it was found that the cleaved form of caspase 3 and 9 in CS group were increasingly expressed as compared to control and such expression was decreased in TMP treated groups (Figure 7(B)). Moreover, Figure 7(C) shows the obvious increase of sperm AR expression after TMP treatments.

### Effect of TMP extracts on TyrPho protein expression in testicular and sperm lysates

**Figure 8.**
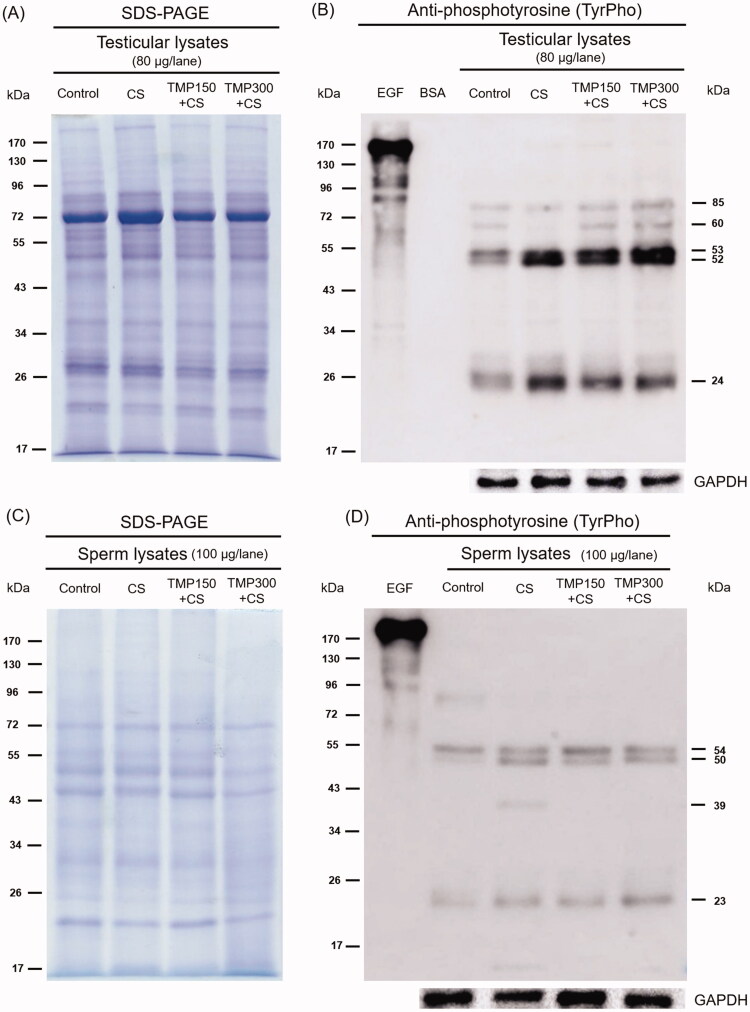
Representative total protein profiles of testis (A) and sperm (C) lysates revealed by SDS-PAGE with their tyrosine phosphorylated (TyrPho) protein expression (B, D). EGF (epidermal growth factor) was used as positive control; BSA (bovine serum albumin) was used as negative control; GAPDH was used as internal control.

As shown in Figure 8(A), the equal protein profiles in testicular lysate of control, CS, TMP150 + CS and TMP300 + CS groups were revealed by using the SDS-PAGE technique for analysing. In testis, five major TyrPho proteins (85, 60, 53, 52 and 24 kDa, respectively) are obviously detected (Figure 8(B)). Compared to control, the expression of 53, 52 and 24 kDa was increased but that of a 60 kDa was nearly absent in CS testis. It was found that TMP could increase the expressions of 85 and 60 kDa TyrPho proteins in CS rats (Figure 8(B)). In addition, the expression of a 24 kDa TyrPho protein was decreased in TMP-CS treated groups as demonstrated in Figure 8(B). The total equal proteins of sperm lysate of all groups are revealed in Figure 8(C). The results showed five major TyrPho proteins (93, 54, 50, 39 and 23 kDa, respectively) present in sperm lysate (Figure 8(D)). Compared to control, the expression of 93 kDa was almost disappeared but those of 50, 39 and 23 kDa were increased in CS rats (Figure 8(D)) It was found that T-MP extract decreased the expressions of sperm TyrPho proteins of 50 and 39 kDa as compared to those from CS group.

## Discussion

Previous studies have demonstrated that CS in male is a cause of significant reductions of androgens, spermatogenesis and sperm quality parameters, causing subfertility or infertility (Song et al. 2016; Starc et al. 2019). It is well known that significantly increased cortisol level in blood serum is a stress marker (Fogelman and Canli 2018). Many reports showed that the CS animal models also possess excess cortisol levels, leading to increase in the reactive oxygen species (ROS) and malondialdehyde (MDA) levels in testis (Jameel et al. 2014; Dutordoir and Bates 2016; Espinoza et al. 2017). Indeed, increased ROS levels are initially involved in apoptosis in testicular germ cells (Dutordoir and Bates 2016; Espinoza et al. 2017; Guoqing et al. 2021). In addition, caspase 3 and 9 proteins are commonly used as potential markers in apoptotic pathways. In this study, the cortisol level and caspase 3/9 expressions were significantly increased in CS group but they were improved in TMP treated groups. Basically, male reproductive organs are androgen dependent organs required for testosterone-AR binding to turn on various functional proteins. Previously, *Mucuna pruriens* (MP) seeds possessing l-DOPA have been shown to stimulate the hypothalamus leading to suppress corticotrophin-releasing hormone (CRH) and adrenocorticotropic hormone (ACTH), resulted in reduced cortisol levels (Suresh et al. 2009; Suresh and Prakash 2012). Generally, a major substance of MP seeds is l-DOPA which has been shown to stimulate the secretion of GnRH leading to increase LH levels (Misra and Wagner 2004; Shukla et al. 2009; Singh et al. 2013; Mutwedu et al. 2019). Therefore, the increased LH could stimulate the secretion of testosterone in Leydig cells. Our TMP seed extract used in this study has been documented to have total phenolic compounds and antioxidant capacity with enhancing of testosterone levels and sperm concentration in adult rats (Iamsaard et al. 2020). Additionally, recently unpublished data showed the presence of l-DOPA in our TMP with high levels revealed by nuclear magnetic resonance (NMR) spectrometer and high-performance liquid chromatography (HPLC) analyses (Supplement data).

CS induction in this study decreased StAR protein expression and testosterone level which was in agreement with previous studies (Lin et al. 2014; Fahim et al. 2019; Guoqing et al. 2021). However, the expression of CYP11A1 was increased in CS group. It was assumed that CYP11A1 attempted to accelerate the conversion to be pregnenolone, major testosterone precursor, to compensate the decreasing of testosterone level. After treated with TMP, the StAR expression and testosterone levels were still not improved but the sperm concentration was corrected, implied for normal spermatogenesis. Previously, the Indian MP has been reported to contain the saponin, a phytoandrogenic compound (Suresh et al. 2013; Anosike et al. 2019). It may act as testosterone to maintain normal sperm production although steroidogenic proteins were not improved. Indeed, the preventive effect on testicular and sperm damages of TMP might come from its antioxidant capacity demonstrated previously (Iamsaard et al. 2020). Basically, AR localized in seminiferous epithelium especially in Sertoli cell is responsible to testosterone binding for essential protein regulations in spermatogenesis (Walker 2010; Roboon et al. 2016; Thanoi et al. 2018). In this study, CS caused germ cell degenerations but Sertoli cells still existed within the seminiferous tubules as demonstrated in histological figures. As equal total proteins subjected in western blot, it could be explained that why the AR expression was increased in CS but it was improved after retrieval of germ cells in TMP treated groups. In addition, antioxidant activity in TMP could also protect the reduction the AR in epididymal sperm. Such capacity might also scavenge the oxidative stress caused from CS, resulted in decreasing of the caspase 9 and 3 expression as demonstrated in this recent study. The presence of testicular Hsp70 has been reported to normally recovery the damaged proteins (Hansen 2014). Hsp70 expression that was increased in TMP treated group was associated with improvement of germ cell epithelium of the thesis. Previously, the testicular and sperm TyrPho expressions have been shown to be altered in CS rats (Arun et al. 2016, 2018, 2021). Numerous TyrPho proteins are involved in sperm production and physiology such as maturation, capacitation and acrosome reaction (Sati et al. 2014). Particularly, TMP could increase the TyrPho proteins at 85 and 60 kDa and improve those of 53, 52 and 24 kDa, indicating functional roles of this extract in testis. Additionally, such changes were also observed in sperm lysate treated with TMP, especially 93, 54, 50, 39 and 23 kDa.

## Conclusions

In conclusion, TMP seed extract possessing l-DOPA and antioxidant activities could improve the sperm qualities and essential testicular proteins under CS condition via enhancing testosterone levels with reduction of apoptotic caspase proteins. The seeds of this plant should be promoted as possible alternative drug or food supplement to increase male reproductive functions under CS condition in male fertility clinic.

## Supplementary Material

Supplemental Material
