# Supplementary material for: Seed extract of Thai Mucuna pruriens reduced male reproductive damage in rats induced by chronic stress
Source: Pharm Biol. 2022 Feb 18;60(1):374–83. doi: 10.1080/13880209.2022.2034896 (PMC8865108; doi:10.1080/13880209.2022.2034896)
Supplement: Supplemental Material [file IPHB_A_2034896_SM7126.docx]

**Supplementary data**

**
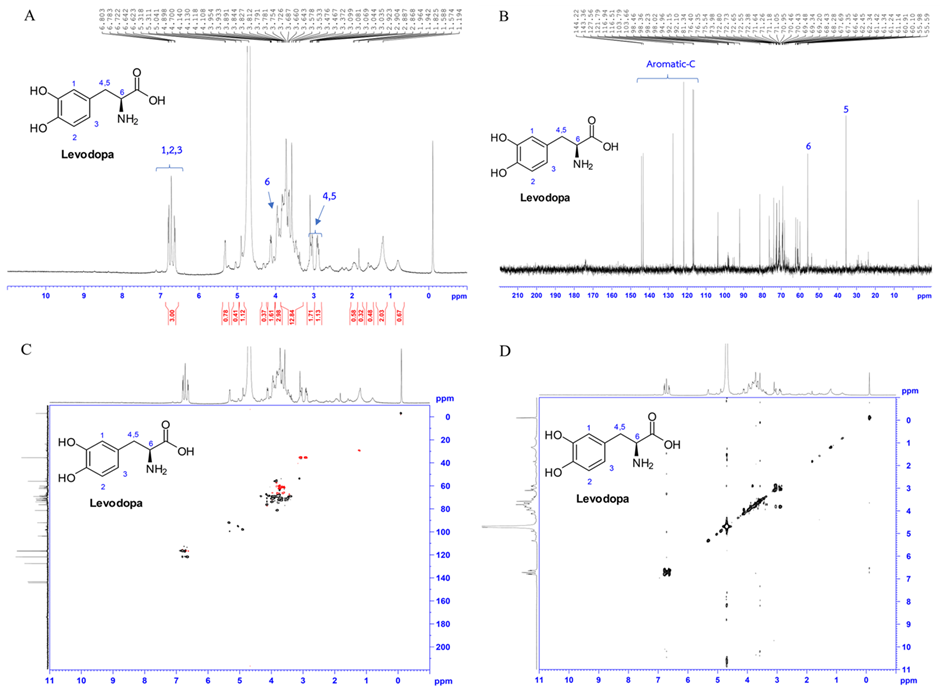
**

**Supplementary Fig. 1.**  Showing nuclear magnetic resonance (NMR) spectrometry of levodopa (L-DOPA) detected in the crude extract of T-MP, demonstrated by ^1^H-NMR (A), ^13^C-NMR (B), ^1^H-^13^C HSQC-NMR (C), and ^1^H-^1^H COSY-NMR (D), respectively.

**
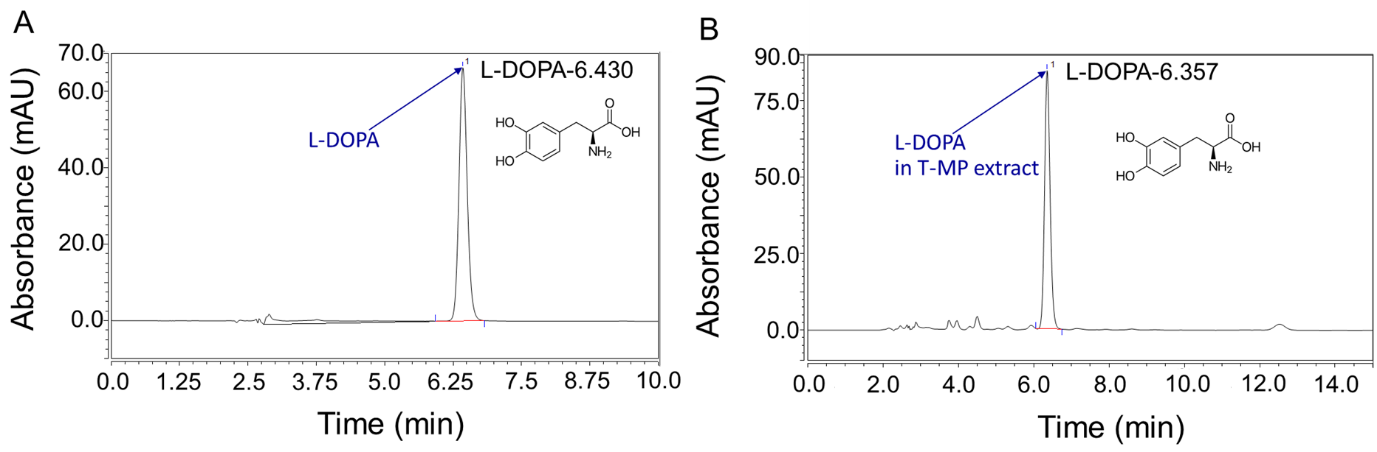
**

**Supplementary Fig. 2** A peak of (A) purified standard levodopa (L-DOPA) and found in (B) T-MP seed extract revealed by high-performance liquid chromatography (HPLC). L-DOPA: Levodopa T-MP: Thai *Mucuna pruriens*.
